# The role of NK cells in fighting the virus infection and sepsis

**DOI:** 10.7150/ijms.59898

**Published:** 2021-07-22

**Authors:** Lu Ma, Qi Li, Suna Cai, Hourong Peng, Ting Huyan, Hui Yang

**Affiliations:** 1 The Hengyang Key Laboratory of Cellular Stress Biology, Institute of Cytology and Genetics, Hengyang Medical School, University of South China, Hengyang, Hunan 421001, China;; 2 School of Life Sciences, Northwestern Polytechnical University, Xi'an, 710072, China.

**Keywords:** Natural killer cells, Viral infection, Sepsis, COVID-19

## Abstract

Natural killer cells, one of the important types of innate immune cells, play a pivotal role in the antiviral process *in vivo.* It has been shown that increasing NK cell activity may promote the alleviation of viral infections, even severe infection-induced sepsis. Given the current state of the novel coronavirus (SARS-CoV-2) global pandemic, clarifying the anti-viral function of NK cells would be helpful for revealing the mechanism of host immune responses and decipher the progression of COVID-19 and providing important clues for combating this pandemic. In this review, we summarize the roles of NK cells in viral infection and sepsis as well as the potential possibilities of NK cell-based immunotherapy for treating COVID-19.

## Introduction

Natural killer (NK) cells are an important kind of innate immune cell and have been defined as “cytotoxic innate lymphoid cells (ILCs) with the capability of 'natural killing' and antibody-dependent cell-mediated cytotoxicity (ADCC)” 1, 2. ILCs are the cells have three main features: absence of recombination activating gene (RAG)-dependent rearranged antigen receptors; lack of myeloid cell and dendritic cell phenotypical markers; and their lymphoid morphology 3. The prototypical ILCs are natural killer (NK) cells and lymphoid tissue-inducer (LTi) cells 4. Based on the distinct patterns of cytokine production, ILCs could be divided into three groups: group 1 ILCs which have the capability to produce interferon-γ (IFN-γ); group 2 ILCs which are able to produce T helper 2 (TH2) cell-associated cytokines, such as interleukin-5 (IL-5) and IL-13; and group 3 ILCs which are capable of producing the TH17 cell-associate cytokines IL-17 and IL-22 4. As the mainly ILCs, NK cells play very important roles in immunity against virus-infected and transformed cells 5, 6. After being discovered in humans and mice in the 1970s, NK cells have received a lot of attention, especially in recent years. In humans, most NK cells circulate in the peripheral blood and represent approximately 15% of peripheral blood mononuclear cells (PBMCs) 7. They are found in other tissues/organs as well, such as the spleen, lymph nodes, thymus, liver (where NK cells represent 50% of resident lymphocytes), skin, uterine decidua and female reproductive tract 8-10.

Traditionally, human NK cells are defined by the expression of CD56 and CD16 and the absence of CD3 (CD56^+^CD16^+^CD3^-^) 11-13. However, NK cells are not a single type of cell, many NK cell subtypes are produced through a complex development process discriminated by the expression levels of particular biomarkers. Accumulating studies outline a linear model of NK cell development 14, which begins with hematopoietic stem cells (HSCs) (CD43^+^) that successively differentiate into lymphoid-primed multipotential progenitors (LPMP) (CD34^+^CD45RA^+^CD10^+^) and common lymphoid progenitors (CLP) (CD34^+^CD45RA^+^CD117), followed by the development of T/NK-committed progenitors with an NK1.1^+^CD117^+^CD44^+^CD25^+^ phenotype. T/NK-committed progenitors can develop into T lymphocytes in a thymic microenvironment; in contrast, when they are co-cultured with bone marrow-derived stromal cells, they can develop into mature NK cells 15. After the acquisition of CD94 expression, T/NK-committed progenitors develop into the CD56^bright^ NK cell subset. Following the downregulation of CD94, acquisition of CD16 and killer immunoglobulin-like receptor (KIR) expression, these cells are defined as the CD56^dim^ NK cell subset 16. In the branch model of NK cell development, LPMPs could differentiate into CLPs or common myeloid progenitors (CMPs), both of which can differentiate into NK cell progenitors 14. CD56^bright^ and CD56^dim^ NK cells are the major subsets of NK cells, among which canonical CD56^dim^ NK cells display higher cytotoxicity toward tumor and infected cells with high perforin and granzyme expression levels 17. Meanwhile, CD56^bright^ NK cells have less cytotoxicity that secrete low levels of perforin and granzymes but produce several cytokines (i.e., IL-5, IL-10, IL-13 and GM-CSF) and chemokines (MIP-1α, MIP-1β, IL-8 and RANTES) and exert an immunoregulatory effect under inflammatory conditions 18-20.

NK cells were presumed to be a relatively homogenous lymphocyte population, particularly compared to T lymphocytes and B cells. However, based on advanced analytical techniques, new evidence has indicated that NK cells actually exhibit a high level of heterogeneity. The data from mass spectrometry/flow cytometry revealed estimated 6,000-30,000 phenotypic populations of NK cells in the peripheral blood of one individual 21. Although researchers have not determined if the numerous phenotypes of NK cells are in mature and stable states or in a transition state of development, it is undeniable that NK cells are highly heterogeneous and play a more complex role in immune regulation than expected.

## NK cells in viral infection

### The strategies of NK cells to discriminate viruses

NK cells are major antiviral lymphocytes that substantially contribute to the host innate immune system and inflammatory cytokine production. In the early phase of infections, NK cells rapidly respond to pathogens before the development of adaptive immune responses 22, 23. The underlying mechanism may relate to the short life span and fast regeneration of NK cells 24. NK cells could be activated in response to infection by different viruses including Zika virus (ZIKV) 25, influenza virus 26, hepatitis C virus 27, dengue virus 28, hantavirus 29, and tick-borne encephalitis virus 30. Biron *et al.* observed increased NK cell proliferation and numbers during an acute infection of lymphocytic choriomeningitis virus (LCMV) in mice 31. Using high-density RNA sequencing, Lum *et al*. confirmed NK cell activation during acute ZIKV infection. ZIKV elicits a robust immune response by NK cells, as evidenced by increased IFN-γ production and CD107a expression 25. In an influenza virus infection model, NK cells produced swift and strong responses in mice, which was proven by the rapid production of type I IFNs and inflammatory cytokines. The rapid NK cell response efficiently controlled early pulmonary viral replication and increased survival 32. These data indicate the important roles of NK cells in antiviral immune responses. Hantavirus infection can cause hemorrhagic fever and strong immune activation, leading a 50% mortality rate 29. Hantavirus could also induce increased NK cell expansion in an IL-15-depended manner. The hyperactivation of NK cells induced by hantavirus may lead to the death of uninfected endothelial cells 29, which indicated the double-faced effect of NK cells on the host response to viral infection. The extensive activation of NK cells has also been observed in response to other viral infections. Activated NK cells could limit viral spread, decrease inflammatory response, and play a protective role in acute viral infection 28, 33.

In humans, NK cells express several activating receptors on their surface, including NKG2D, 2B4, NKp80, NKp30, NKp44, NKp46, etc., while 2B4 and NKR-P1C (NK1.1) are the activating receptors in mice 34. Relying on these receptors, NK cells could rapidly recognize and kill malignant cells and virus-infected cells. Meanwhile, inhibitory receptors, including KIRs, killer cell lectin-like receptor G1 (KLRG1), members of the leukocyte immunoglobulin-like receptor (LIR) family and the NKG2A-CD94 receptor complex expressed in human NK cells and Ly49A/C/I/P expressed in mice, maintain autoimmune tolerance by binding to human leukocyte antigen (HLA), self-MHC-I molecules and related proteins or classical cadherins, (E-, N-, and R-cadherins) 34-36. In addition, other inhibitory receptors, including CEACAM1, CD300a, and TIGIT in human NK cells, can discriminate non-MHC-I ligands, which are important for maintaining NK cell homeostasis 37-41.

Whether NK cells trigger cytotoxicity depends on the delicate balance between inhibitory and activating signals. Natural cytotoxicity receptors (NCR), including NKp30, NKp44 and NKp46, are the dominant activating receptors in NK cells identifying virus-infected cells 38, 42. After binding to their ligands, activating receptors recruit immunoreceptors such as tyrosine-based activating motif (ITAM)-containing adapters, including DAP10, DAP12, FcεRIγ and CD3ζ, to transmit downstream signals through tyrosine kinases and induce the cytotoxicity of NK cells 38. When activating signals overpower inhibitory signals, NK cells will kill target cells. In addition, activating receptors are also required for NK cell expansion during viral infection 43. Influenza virus hemagglutinin (HA) is the first recognized ligand of NCR 38, and poxvirus HA can be recognized by NKp30 and NKp46 44. The HA-neuraminidase (HN) of Sendai virus, paramyxoviruses, avian Newcastle disease virus and human parainfluenza virus 3 (HPIV3) is the ligand of NKp44 and NKp46 38, 45-47. NKp44 also recognizes the E-protein of dengue virus and West Nile virus 48. Additionally to directly distinguish viral proteins, NK cells also distinguish the upregulated NKp44L in host cells caused viral infection such as poxviruses, herpesviruses and HIV to trigger cytotoxicity 49-51. In addition to NCRs, it was recently proven that Toll-like receptor (TLR) can be employed by NK cells to engage pathogen-associated molecular patterns (PAMPs), such as bacteria-associated peptidoglycan, LPS, virus-derived dsRNA and specific DNA with CpG motifs 52. For example, TLR2 is critical for NK cell activation in response to vaccinia virus (VV) infection by activating the TLR2-MyD88 signaling pathway 53. TLR3 and TLR4 can recognize mouse-adapted SARS-CoV and induce inflammatory reactions through MyD88 54 and TRIF-mediated pathways 55. Therefore, it is worth studying whether there is an analogous recognition pattern between SARS-CoV-2 and host immunocytes.

NKG2D is the receptor that NK cells used to recognize transformed cells and plays an important role in anti-tumor immunological surveillance 56. It is also a key player in NK cell-mediated cytotoxicity during viral infection. Rather than directly recognizing viral antigens, NKG2D recognizes various MHC I-like ligands, which are often downregulated in virus-infected cells 56, 57. For example, Kaposi's sarcoma (KS)-associated herpesvirus (KSHV) downregulates the expression of MHC class I on KSHV-infected cells to avoid being recognized by MHC-restricted CD8^+^ T cells, which renders these cells susceptible to being killed by NK cells through the induction of NKG2D-mediated activating signaling 58. UL16-binding protein (ULBP), which binds to the human cytomegalovirus (HCMV) glycoprotein UL16, is another key ligand of NKG2D 59. After HCMV infection, ULBPs expressed by infected cells interact with NKG2D/DAP10 to trigger NK cell-mediated cytotoxicity 60. Each NK cell receptor interacts with several distinct ligands, but most NKG2D ligands are still unknown 56, 61. DNAM-1 (CD226) is another important NK cell receptor that mediates anti-tumor immunity 62; it is also important for NK cells to discriminate viruses. The known ligands of DNAM-1 are poliovirus receptor (PVR) (CD155) and Nectin-2 (CD112), which are latent entry receptors of several viruses to invade cells 63, 64. Similar to NKG2D, DNAM-1 discriminates ligands on virus-infected cells and triggers NK cell cytotoxicity. TIGIT is a receptor from the same immunoglobulin-like superfamily as DNAM-1, but it exerts inhibitory effects on NK cells 65. TIGIT binds to the same ligands as DNAM-1 on target cells through a competitive interaction and counteracts NK cell activation 66.

**Figure 1:**
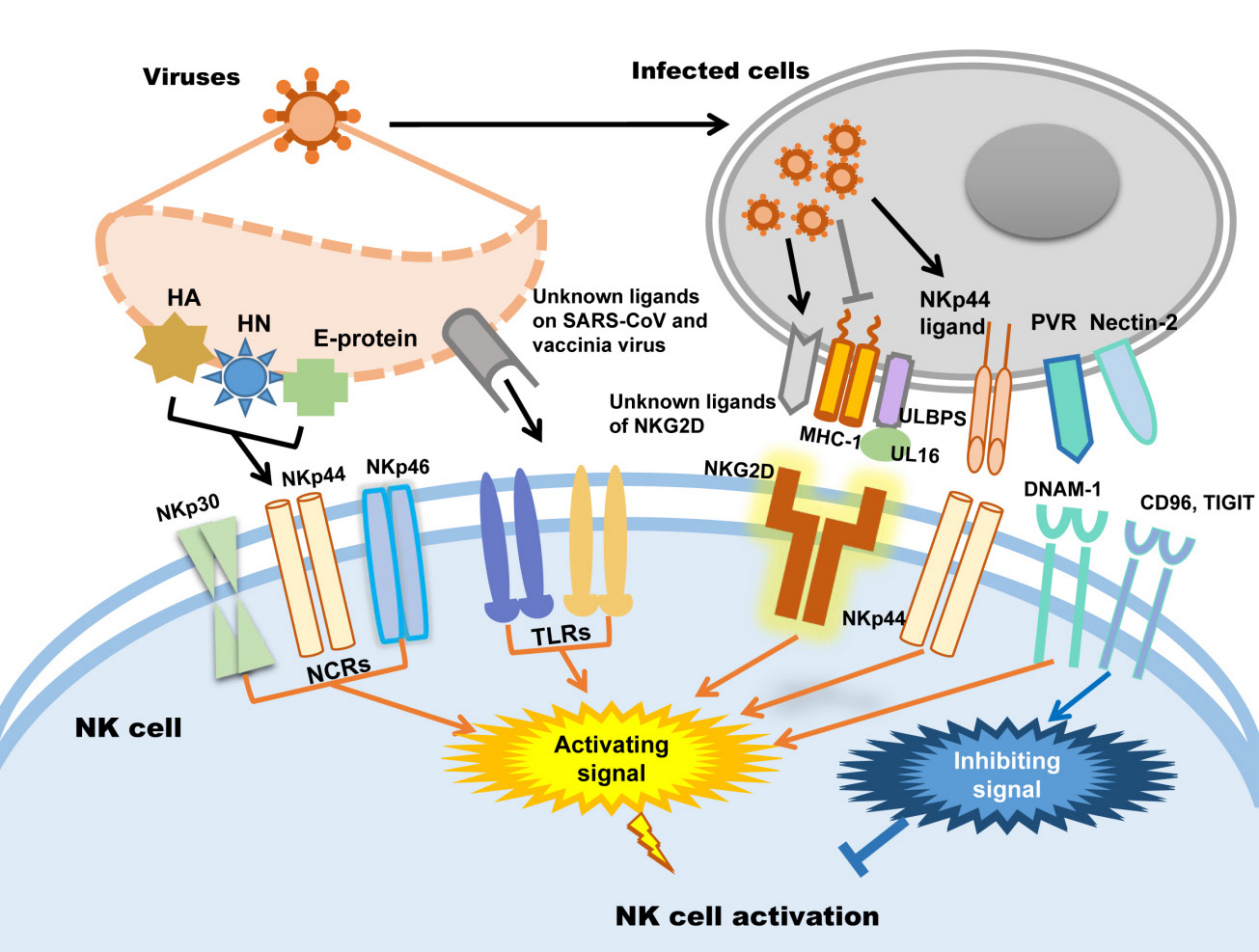
NK cell activation during viral infection.

To sum up, there are mainly two strategies of NK cells to be activated in viral infection: one is NK cells activating receptors distinguish the viral protein such as HA, HN and E-protein directly; the other way is the activating receptors distinguish the changed expression of MHC-1 and ULPBs on virus infected cells. Subsequently, the activating signal could be transmitted by activating receptors to trigger NK cells cytotoxicity. The patterns of NK cell activation in viral infection are illustrated in Figure 1.

### Viral mechanisms to escape NK cell surveillance

During the constant battle with the host immune system, viruses have evolved multiple strategies to evade elimination and induce chronic infection. Several viruses, including HCMV 67, 68, mouse CMV 69, 70, zoonotic orthopoxviruses 71, and HIV 72, escape NK cell-mediated elimination by downregulating the expression of NKG2D ligands. For instance, HCMV downregulates the expression of the NKG2D ligands ULBP1, ULBP2, MICB and MICA on infected cells by expressing the viral glycoproteins UL16 and UL142 68, 73. Similarly, in individuals with acquired immune deficiency syndrome (AIDS), HIV-1 decreases the cell surface expression of MICA, ULBP1 and ULBP2 in infected cells through Nef protein, which decreases the susceptibility of the virus to NK cell-mediated lysis 72. KSHV downregulates NKp44L expression on infected cells through the ORF54 gene-encoded protein to escape NK cell-mediated killing 74. Furthermore, viruses have developed another strategy, upregulating the expression of inhibitor receptor ligands to block NK cell activity. For instance, through the activation of the RIGI-IRF3 pathway mediated by IFN-β, ZIKV increases the expression of MHC-I molecules on infected cells 75. As mediated by the viral protein MATp1, mouse cytomegalovirus (MCMV) rescues the expression of some MHC-I molecules on infected cells, which are engaged by the inhibitory receptor Ly49. The rescued self MHC-I molecules exhibit increased affinity for Ly49 and inhibit signaling in NK cells 76.

In addition to downregulating the ligands of NK cell-activating receptors and upregulating the ligands of NK cell-inhibitory receptors on infected cells, the viruses, particularly those readily induce chronic infections, directly impair NK cell cytotoxicity by altering their phenotypes and functions. For example, in chronic hepatitis virus infection, hepatitis C virus (HCV) downregulates NKG2D expression on circulating NK cells through an NS5A-mediated pathway and downregulates NKp30 expression by increasing the levels of an antagonistic NKp30 ligand on HCV-infected cells, which subsequently impairs NK cell-mediated cytotoxicity, ADCC and IFN-γ and TNF-α production 77, 78. In the same way, during chronic hepatitis B virus (HBV) infection, HBV releases the antigens HBsAg and HBeAg, which directly block NK cell activation, cytokine production and cytotoxic granule release by suppressing the STAT1, NF-κB and p38 MAPK pathways 79. 2B4 is a CD2-related receptor belonging to the signaling lymphocyte activation molecule (SLAM) family 80, that is expressed by NK cells, γδT cells, basophils, monocytes and a subset of CD8^+^αβ T lymphocytes 81. In patients with persistent HBV infection, HBV downregulates the expression of NKG2D and 2B4 on NK cells by increasing TGF-β1 expression, subsequently impairing NK cell-mediated cytotoxicity and IFN-γ production 82. In another strategy, human T-cell leukemia virus type 1 (HTLV-1) downregulates the expression of intercellular adhesion molecule 1 (ICAM-1) and ICAM-2 on infected CD4^+^ T cells, which subsequently prevents NK cells from adhering to HTLV-1-infected cells and prevents NK cell-mediated death 83. Furthermore, Japanese encephalitis virus (JEV) inhibits NK cell proliferation by inducing endothelial cell shedding of sHLA-E, which inhibits IL-2- and PMA-mediated ERK 1/2 phosphorylation in NK cell lines 84. Viruses can also activate immunosuppressive cells, such as myeloid-derived suppressor cells (MDSCs), to impair NK cell activity and block IFN-γ production 85.

### Viral infection-related cytokines and signaling pathways in NK cells

Cytokines and signaling molecules play important roles in the activity and function of NK cells under physiological and pathological conditions. Specifically, IL-2, IL-12, IL-15 86, IL-18, and IFN-γ are the most important cytokines that are secreted by immunocytes and infected tissues 87 to regulate NK cell-mediated cytotoxicity 86. For example, in VV infection, efficient NK cell activation depends on dendritic cells (DCs) and IL-18 signaling, as well as the TLR2-MyD88 signaling pathway 53, 88. Meanwhile, both intrinsic and extrinsic STAT1 signaling are indispensable in NK cells responding to VV infection 89. Furthermore, integrin α2β1 dimers and IFN-α are required for optimal NK cell expansion during viral infection 90, 91. IFN-α induces the expression of its downstream transcription factors, including STAT1, STAT2 and IRF9, and these transcription factors can induce the transcription of hundreds of IFN-stimulated genes and the “antiviral state” in NK cells 92. Recently, adrenergic signaling has been proposed to play a novel role in regulating circulating lymphocytes responding to viral infections 93. Diaz-Salazar *et al*. reported that depending on IL-12 and STAT4 signaling, NK cells upregulate *Adrb2* (which encodes the β2-adrenergic receptor) to maintain their proliferative capacity during MCMV challenge 93. It is beneficial to decipher the underlying complicated signaling networks of NK cell activation for developing strategies to manipulate NK cell function under viral infection, control viral replication in the early stage and prevent severe inflammatory reactions, even sepsis.

Individuals experiencing severe viral infection caused by herpes virus 94, respiratory syncytial virus 95, HCMV, Epstein-Barr virus (EBV), herpes simplex virus (HSV), HBV, HCV and HIV 33, 96 often show impaired NK cell functions. However, NK cells can be a double-edged sword by exerting the unwanted effect of tissue damage in severely infected patients. Fu *et al.* found that NK cells can mediate hypersensitivity and the pathogenesis of HCV-induced liver injury in mice 97. By blocking NKG2D and ligand interactions, liver injury was completely prevented in mice model of hepatitis 98. In a model of an acute viral infection of the central nervous system (CNS), mice deficient in NK cell-mediated cytotoxicity were more resistant to a lethal virulent Semliki Forest virus (vSFV) infection than wild-type mice, suggesting that the cytolytic activity of NK cells may be detrimental under specific circumstances 99.

In conclusion, multiple studies have revealed the intricate functions of NK cells in the immune response to viral infection. Undoubtedly, the expression levels of various NK receptors play important roles in the differential dynamics of NK cell activation and the viral infection process. Intranasal mouse hepatitis virus type 1 (MHV-1) is a group 2 respiratory CoV, and its intranasal infection induces a lung pathology in mice which is similar to the pathological state of patients with severe acute respiratory syndrome (SARS). Using this model, Khanolkar *et al*. found the contributions of NK cells and type I IFN-mediated signaling to reducing morbidity and mortality of MHV-1-infected mice 100. In mice infected with mouse hepatitis virus type 3 (MHV3), a coronavirus, the production of NK cells was significantly impaired, which induced the occurrence of fulminant hepatitis 101. In another study, pigs were pre-infected with porcine reproductive and respiratory syndrome virus (PRRSV) to simulate immunosuppressive respiratory disease. Following repeated infection with porcine respiratory coronavirus (PRCV), significant reductions in innate NK cell-mediated cytotoxic functions were observed in PRRSV and PRCV co-infected pigs 102. These results indicated the potential role of NK cells in the host immune response to combat CoV infections 100, 102.

## NK cells in sepsis

Sepsis is a pathological process that is induced by a severe systemic infection accompanied by a dysregulated immune response and overexpression of inflammatory factors, which exerts substantial negative effects on health, including damage to multiple organs 103, 104. Severe sepsis will induce hypotension and hypoperfusion, and cause lactic acidosis, oliguria, and acute respiratory distress syndrome (ARDS) 105, resulting in an approximately 26% mortality rate 106.

Many pathogens including bacteria, fungi and viruses can induce sepsis; however, more than 70% of sepsis cases are caused by bacterial infections 107, 108. There are no definite diagnostic criteria for discriminating viral sepsis and bacterial sepsis to date, which may lead to unnecessary antimicrobial use 107. Sporadic studies indicated that elevated procalcitonin levels are more relevant to bacterial infections than viral infections, though related immunological profile data are still scarce for viral sepsis 107, 109. Except for the well-known overactivation of the immune system during the beginning of sepsis, sepsis-induced immunosuppression cannot be ignored. It was shown that there is a decreased quantity or increased apoptosis of T and NK cells accompanied by reduced INF-γ production in the 'late phase' of sepsis, which can cause secondary infection and even death 110, 111.

Relying on the recognition of different PAMPs by TLRs and NCRs 52, 112, 113, NK cells play very complicated role in sepsis. TLR-4 (Toll-like receptor 4) is a known receptor that recognizes lipopolysaccharide (LPS) from gram-negative bacteria. NK cells display low TLR-4 expression on their surface and moderate TLR4 expression in the cells, which can be activated by PAMPs to produce IFN-γ 114. In a *Pseudomonas aeruginosa-*induced mouse pneumonia model, splenic NK cell populations were increased accompanied by increased IFN-γ secretion 115. In another mouse model of sepsis that formed by cecal ligation and puncture (CLP) followed by LPS injection, the number of NK cells with low IL-18R expression levels was increased in the mouse liver 116. Mouse models depleted of NK cells are ideal tools to study NK cell function. In mice infected with pulmonary nontuberculous mycobacteria (NTM), NK cell depletion increased the bacterial load and mortality rate 117. However, in another study, compared to their wild-type (WT) counterparts, NK cell-deficient mice (IL-15^-/-^ mice) showed higher survival rates and lower levels of pro-inflammatory cytokines 118.

As the main killing proteins secreted by NK cells, the role of granzymes in sepsis should not be ignored. Granzyme-deficient mice (both gzmA^-/-^ and gzmM^-/-^) showed decreased production of pro-inflammatory cytokines compared to WT mice, which are unlikely to develop endotoxic shock 119. In addition to their cytotoxic effects, granzymes play an important role in regulating the secretion of pro-inflammatory cytokines 120, 121, and this may exacerbate LPS- or endotoxin-mediated cytokine secretion during endotoxic shock 122.

Animal model-based findings revealed the complicated roles of NK cells in the immunopathogenesis of sepsis. However, the existed experiments had produced contradictory results 123, 124. There are complex reciprocal regulatory pathways between NK cells and other immune cells, including DCs, macrophages, and neutrophils, as well as several cytokines involved during the sepsis process. Researchers have not been able to determine whether NK cells exert a positive or negative effect on sepsis. All the data should be interpreted carefully because of the heterogeneity between mouse models and humans as well as between mouse models themselves.

In a clinical study, unfavorable outcomes including death were observed in patients with severe sepsis whose circulating NK cell number was less than 20% of the total lymphocyte population. An early increase in the circulating NK cell population will increase the survival rate of patients 125. In another extensive clinical trial, Gogos *et al*. found that patients with sepsis have a significantly lower number of circulating NK cells than patients with community-acquired pneumonia (CAP) 126. According to Forel *et al*., the number of NK cells (CD56^+^CD3^-^) in blood samples collected from patients in the ICU is significantly reduced during all stages of sepsis and shows indiscriminate features such as degranulation, as indicated by CD107 or LAMP-1 (lysosomal-associated membrane protein-1) expression and decreased cytotoxicity compared to NK cells from healthy controls 127. Furthermore, data from an antibody-dependent cell cytotoxicity (ADCC) assay showed that NK cells from patients with sepsis secrete low levels of IFN-γ compared with those from their healthy counterparts 127.

**Figure 2:**
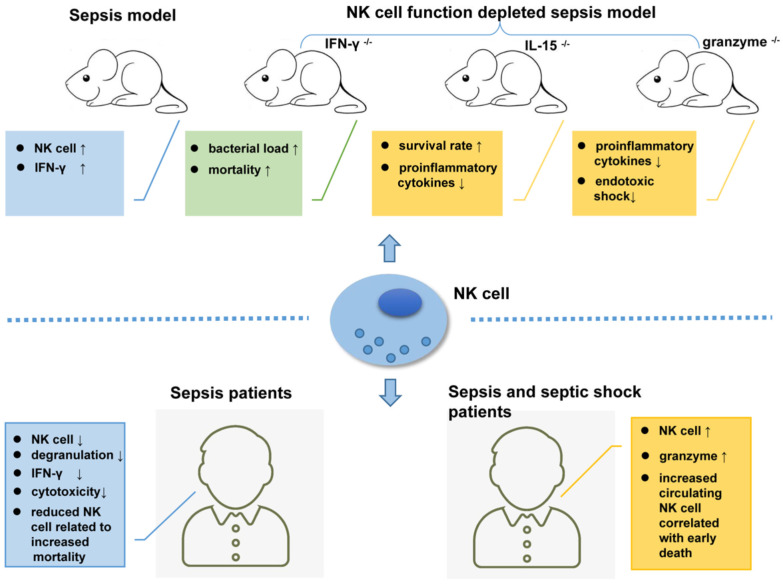
NK cells are key players in the immunopathogenesis of sepsis patients and mouse models.

In addition to the decreased number, impaired NK cell function has also been observed in clinical studies. As shown in the study by Feng *et al*., NK cell cytotoxicity dramatically decreased during sepsis, which may result from reduced CD3^-^CD56^+^ NK cell cluster differentiation, a shift in the phenotype of NK-activating receptors toward inhibitory receptors, and impaired cytokine production in septic patients 128. Consequently, the phenotypic changes and impaired functions of NK cells might be one of the underlying causes of immunosuppression during sepsis 128. Nevertheless, another study reported inconsistent results, in which NK cells isolated from patients with sepsis released larger amounts of IFN-γ compared to healthy controls after treatment with LPS 129. Furthermore, Andaluz-Ojeda *et al.* found that an increased number of circulating NK cells (> 83 cells/mm^3^) is correlated with early death in patients with sepsis 130. The levels of granzyme proteins (granzyme A, granzyme B, and granzyme K) are all increased in NK cells from patients experiencing sepsis and septic shock who suffer from multiple organ dysfunction, with an increased mortality rate 131-133. Thus, plasma granzyme levels may be a potential biomarker determining the severity of sepsis 104. Figure 2 shows the complex roles of NK cells in sepsis.

The significant similarities of immune profile between sepsis and COVID-19 have been reported by Lopez-Collazo et al. 134, or in other words SARS-CoV-2 may be one of the etiological agent causative of sepsis 135. The common symptoms of sepsis and COVID-19 include excessive inflammation and cytokine storms 136, chronic basal inflammation state 136, high granulocyte-macrophage (GM)-CSF levels in circulating lymphocyte populations, excessive macrophage activation, depletion of lymphocytes, as well as the overexpression of some immune checkpoints 134. C5a is an inflammatory protein, which can bind to the immunocytes expressing C5aR including NK cells to induce the production of various cytokines and chemokines 137. A recent study showed that the serum levels of soluble C5a are increased in SARS-CoV-2 patients 138, which indicate the NK cells may involve in the SARS-CoV-2 induced cytokine storm by C5a regulation. However, another latest study based on single cell sequencing indicated that peripheral monocytes may not contribute to the putative cytokine storm in COVID-19 139.

The exact role of NK cell in sepsis is very difficult to interpret clearly. First, researchers have not been able to determine whether NK cell dysfunction is a cause or a consequence of sepsis. When other factors induce NK cell dysfunction, exhausted NK cells may not control the progression of sepsis timely and can reverse patient deterioration, or the terrible pathological conditions in sepsis patients could induce NK cell dysfunction. Analogously, hyperactivation of NK cells may be interpreted as the cause of sepsis or the compensatory phenomenon because NK cells actually control sepsis effectively. Although specific effects of NK cells on viral infection and sepsis have not been clearly elucidated, we can still make a bold assumption that highly active NK cells may effectively control viral infection in the early stage by directly killing infected cells or promoting the infiltration of other immune cells, such as neutrophils, T lymphocytes and B cells, into the lesion. However, if the initial infection is not controlled timely and effectively, the hyperactivated NK cells will produce excessive levels of pro-inflammatory factors by releasing granzymes, subsequently causing organ injury and even death. Without a doubt, this assumption needs more evidence to be proven or disproven. Additional carefully designed studies should be performed in future to further elucidate the exact role of NK cells in sepsis. For example, if the feasibility and difficulty are not being regarded, the NK cell profile, including the quantity, activity and the ability of NK cells to produce cytokines and cytotoxic granules as well as their specific subtypes, should be monitored constantly in patients with a severe infection and even sepsis. More importantly, clinicians should monitor and analyze the changes in other immunocytes and the complex interactions of each type of immunocyte in the pathological process of sepsis. Single-cell sequencing, a state-of-the-art method with apparent advantages, would be a powerful tool for decoding the specific functions of NK cells and complex mutual regulation with other immune cells in patients with sepsis.

## NK cell-based immunotherapy for viral infection

The pivotal role of NK cell in monitoring and controlling tumors has been extensively confirmed, and NK cell-based tumor immunotherapy has been extensively developed, including autogenous or allogeneic NK cell expansion, gene modification of NK cells and various chimeric antigen receptor (CAR) NK technologies (reviewed by Fang *et al*.) 140. Accordingly, NK cell-based immunotherapy is presumptive to be effective at controlling and relieving infections. Because interleukins exerting a positive regulatory effect on NK cells, it may be worth determining whether they could increase NK cell activity during severe infection. Studies have indicated that IL-23 is beneficial for reverting sepsis-associated immunosuppression by activating both NK cells and DCs, and further stimulating protective T cell immune responses 141, 142. Additionally, IL-12 and IL-27 may have analogous therapeutic potential with IL-23 for alleviating sepsis 142, 143. PD-1 (programmed death receptor-1), also named CD279, is a well-known immune checkpoint that is mainly expressed on T lymphocytes and NK cells. By initiating lymphocyte apoptosis, PD-1 induces immunosuppression and prevents hyperactivation of lymphocytes 144. Both PD-1 and its ligand PD-L1 (programmed death receptor ligand 1) are highly expressed on lymphocytes from patients with sepsis, which may partially explain the sepsis-associated immunosuppression and increased mortality 145-146. Therefore, PD-1 antibodies have been used to treat sepsis in mouse models. Shindo *et al*. found that anti-PD-L1 peptide compound 8 treatment doubled the survival rate of mice with sepsis 148. Chang *et al*. and Patera *et al*. assessed the therapeutic effects of anti-PD-1 and anti-PD-L1 antibodies separately by isolating and culturing lymphocytes from septic patients *in vitro.* These antibodies increased the survival rate of immunocytes and the production of IFN-γ and IL-2 by NK cells 145, 149. Corresponding clinical experiments are in progress (ClinicalTrial.gov# NCT02576457). In addition to the PD-1/PD-L1 recognition system, other immune checkpoints, such as cytotoxic T lymphocyte antigen-4 (CTLA-4), T cell membrane protein-3 (TIM-3), lymphocyte activation-gene-3 (LAG-3) and 2B4, are upregulated during the course of sepsis 150. Studies focused on the therapeutic effects of these immune checkpoints on sepsis have been conducted in mouse models 151, 152, and clinical trials have shown promising results 153. Therefore, the specific inhibitors or antibodies targeting immune checkpoints may be ideal candidates for ameliorating and treating sepsis.

CAR is a strategy in which immune cells (T lymphocytes or NK cells) were engineered by gene fusion and transfection to express a CAR protein that constitutes an antigen-binding region (scFv), transmembrane region and signal transduction region. The scFv fragment is extracellularly presented to recognize and bind tumor antigens, and the intracellular signal transduction region transmits activating signals in immunocytes 154. CAR therapy significantly improves the killing effects of immune cells on tumors and has been used in tumor immunotherapy clinically. In addition to tumor therapy, CAR-T cell-based treatments targeting severe viral infections, such as HIV in AIDS [155-159, ClinicalTrial.gov# NCT03240328 and NCT01013415], HBV infection 160-162 and infection with HCMV 163, have been developed and analyzed in animal models and clinical trials. In an *in vitro* trial, canine NK cells isolated from PBMCs of normal dogs were expanded; these cells produced large amounts of IFN-γ and exhibited dose-dependent cytotoxicity toward canine distemper virus (CDV)-infected Vero cells. Pretreatment with anti-CDV serum from hyperimmunized dogs enhanced the ADCC of NK cells against CDV-infected Vero cells. These results emphasize the potential effect of expanded NK cells for treating CDV infection 164. All of these examples illuminate the possibility of using NK cell-based immunotherapy strategies, including *in vitro* amplification and each type of CAR method, for treating severe infections and even sepsis. To date, NK cell or CAR-NK cell-based immunotherapy for viral infection are emerging gradually and the related clinical trials were summarized in Table 1.

## Fighting COVID-19, do NK cells provide opportunities?

At the end of 2019, the novel coronavirus (SARS-CoV-2) emerged and has rapidly spread across the world. The COVID-19 has killed more than three million individuals globally till April 2021. In China, the mortality rate is substantially increased to 6.4% when the patient is greater than 60 years old compared to lower than 1% in young people 165. When the patient is aged 80 years or older, the mortality rate increases to 18.4% 165. It has been proven that aging can induce immune dysregulation by decreasing cell-mediated immune function and humoral immune responses 166-168. Specifically, with aging, NK cells showed the profile of decreasing of immature CD56^bright^ NK cells and the increasing of highly differentiated CD56^dim^CD57^+^ NK cells 169, 170, as well as downregulation of NKG2A 171 and concomitant the upregulation of KIR family members 172. This phenomenon indicated the impaired proliferation ability and increased cytotoxic capacity and ADCC of NK cells in elder people 173. Therefore, the role of NK cells in significant correlation between aging and death in COVID-19 is still waiting to be revealed. Other factors that significantly increasing the mortality rate are various basic diseases, including hypertension, diabetes, respiratory system, renal and lung diseases 174. The main causes of death include excessive inflammation induced by a proinflammatory cytokine storm, disseminated intravascular coagulation (DIC) and thrombus-induced pulmonary dysfunction that subsequently induce ARDS 174 and multiple organ dysfunction (MODS) 175. Similar to SARS and MERS (Middle East respiratory syndrome coronavirus) 176, a reduced number of lymphocytes, particularly CD4^+^CD8^+^ T lymphocytes, has been detected in patients during the early stage of COVID-19; reduced lymphocytes are also an important signal predicting disease severity 177, 178.

Clinical study reported that NK and CD8^+^ T cells were both found to be markedly decreased in patients with SARS-CoV-2 infection 179, 180. In addition, asymptomatic patients showed higher counts of lymphocytes, T cells, B cells, and NK cells compared to the symptomatic COVID-19 patients 181, 182. Specifically, NK and CD8^+^ T cell activity was impaired by overexpression of the inhibitory receptor NKG2A in COVID-19 patients 179, 180. In a latest studies, NK cells were proved activated across distinct subsets in peripheral blood of COVID-19 patients by using multi-color flow cytometry and single-cell RNA sequencing, which was hallmarked by high expression of perforin, NKG2C, and Ksp37 183. However, another study using single-cell sequencing indicated that both CD56^dim^ and CD56^bright^ NK cells were depleted in COVID-19 samples as well as NK cells appeared exhausted based on expression of LAG3, PDCD1 and HAVCR2 in patients with COVID-19 139. The in vitro study indicated the SARS-CoV-2 can induce NK cells exhaustion via Spike 1 protein binding to the HLA-E of lung epithelial cells and trigger HLA-E/NKG2A pathway 184. These results indicate the important role of NK cells in pathological COVID-19 processes. Nevertheless, more studies focusing on the role of NK cells in SARS-CoV-2 infection are urgently needed, which will be beneficial for developing effective countermeasures for SARS-CoV-2 infection.

Last but not least, few pioneering clinical trials using NK cells to treat COVID-19 patients are ongoing (ClinicalTrial.gov# NCT04344548, NCT04365101, NCT04280224), as well as an NKG2D-ACE2 CAR-NK based trail (ClinicalTrial.gov# NCT04324996). These works will be expected to give direct and strong evidence on the effect of NK cell therapy in combating COVID-19. However, some issues should be considered; for example, when is the optimum time to administer NK cells to patients? If uncontrolled inflammation has occurred, the increased NK cells and their hyperactivation may further exacerbate the inflammatory response and cause more damage. The immune environment and cytokine milieu in patient are unique, which may induce unknown and uncontrollable immune reaction of NK cells. Additionally, NK cells from COVID-19 patients must be utilized in biosafety level 3 facilities, which are rare, and the operation may increase the infection risk to operators.

Another strategy is to develop an NK cell-based vaccine targeting SARS-CoV-2. Memory is one of feature of adaptive immune responses of antigen-specific T and B lymphocytes, which provides the ability to evoke a rapid and effective response to secondary infections 185. The 'memory function of NK cells' is a concept that has only been proposed recently, stating that NK cells have memory-like, antigen-specific, long-lived adaptive immune responses 186, 187. Evidence shows that in T- and B-cell-deficient mice, adoptive transfer of virus-sensitized hepatic NK cells into naive recipient mice enhanced the survival of the mice after lethal challenge with the same sensitizing virus 188. In humans, there are epigenetic modifications and antibody-dependent expansion of memory-like NK cells in HCMV-infected individuals 189. Likewise, NK cells from CMV- and EBV-infected individuals have the ability to recognize autologous B cells loaded with virus-derived peptides and exhibit antigen-specific cytotoxicity 190. Based on these findings, it is worth determining whether administration of pre-expanded autogenous NK cells challenged with inactivated SARS-CoV-2 or its spike protein provide a specific amount of immunity and protection. More studies are needed to elucidate the pathology of COVID-19 and the interaction of SARS-CoV-2 with immunocytes. Animal models that reproduce the clinical features of COVID-19 are developing fast, which might significantly help people to combat this disease 191.

## Figures and Tables

**Table 1:** NK cell-based clinical trials in CAR NK cells from ClinicalTrial.gov (Last accessed: April 28, 2021)

Conditions	Therapeutic(s)	Phase	Patients	Trial identififier	Status
HIV Infections	Haploidentical NK cell; N-803	Phase 1	9	NCT03899480	Completed
HIV	NK cells; IL-2	Phase 1	4	NCT03346499	Completed
Corona Virus Infection	T memory cells; NK cells	Phase 1|Phase 2	58	NCT04578210	Recruiting
SARS-CoV-2	CYNK-001 cells	Phase 1|Phase 2	86	NCT04365101	Recruiting
CMV Viremia|Transplantation Infection	expanded NK cells	Not available	10	NCT04320303	Recruiting
Novel Coronavirus Pneumonia	NK Cells	Phase 1	30	NCT04280224	Recruiting
Covid19	Off-the-shelf NK Cells (KDS-1000)	Phase 1|Phase 2	54	NCT04797975	Not yet recruiting
Covid19|Sars-cov 2	atural Killer Cells infusion	Phase 1	24	NCT04634370	Not yet recruiting
COVID	Allogeneic NK transfer	Phase 1|Phase 2	10	NCT04344548	Not yet recruiting
COVID-19	NK cells,IL15-NK cells,NKG2D CAR-NK cells,ACE2 CAR-NK cells,NKG2D-ACE2 CAR-NK cells	Phase 1|Phase 2	90	NCT04324996	Recruiting
